# The dead and the dying - a difficult part of EMS transport: A Swiss cross-sectional study

**DOI:** 10.1371/journal.pone.0191879

**Published:** 2018-02-27

**Authors:** Rebecca Maria Hasler, Sandra Stucky, Heinz Bähler, Aristomenis K. Exadaktylos, Frank Neff

**Affiliations:** 1 Department of Emergency Medicine, Inselspital, University Hospital Bern, Bern, Switzerland; 2 Sanitätspolizei Bern, Emergency Medical Service Bern, Bern, Switzerland; 3 Department of Anesthesiology and Pain Therapy, Inselspital, University Hospital Bern, Bern, Switzerland; Azienda Ospedaliero Universitaria Careggi, ITALY

## Abstract

**Objective:**

Most deaths occur in the pre-hospital setting, whereas mortality in the emergency department (ED) is low (<1%). However, our clinical impression is that some patients are being transported to hospital in devastating conditions with no likelihood of survival, but demanding extensive hospital resources. The decision on whether to transport a dying person to hospital or not is a difficult task for emergency medical services (EMS) personnel. As there is little epidemiological data about these patients, this paper aims to describe this special population.

**Methods:**

Retrospective cross-sectional study on adult patients transported by ground ambulance to the ED of a Swiss university hospital, who died during their stay in the ED between January 2008 and December 2012. Data was collected on the basis of ambulance report forms and discharge summaries of the ED.

**Results:**

One hundred and sixty-one patients were analysed. Most deaths were due to cardiovascular diseases (43%). Only 9% of patients died of trauma. The median age was 70 years (IQR 56–81 years) and 70% (n = 112) were men. Trauma patients were significantly younger (median age 55 years, p<0.001). The overall mortality rate was 0.9% for all patients transported by EMS to the ED. About one third of all patients received cardiopulmonary resuscitation (CPR) from bystanders (n = 53). The most common electrocardiogram (ECG) findings were asystole (n = 57) and pulseless electrical activity (n = 91). Fifty percent (n = 64) of the resuscitated patients were defibrillated. Three quarters (n = 115, 72%) of all patients were intubated on site. The mechanical chest compression device Lucas™2 was mainly used in cases of cardiovascular or uncertain cause of death and did not reduce the operating time on site.

**Conclusion:**

The low ED mortality rate of 0.9% shows that only a few dying patients are transported to hospital. However, transport to hospital has to be carefully evaluated, especially for elderly patients with asystole or PEA due to medical conditions. The low CPR rate from bystanders demonstrates that public CPR training should be promoted further. The use of Lucas™2 did not reduce the operating time on site. For further investigations, comparison with survivors would be needed.

## Introduction

Death is a daily topic for emergency medical services (EMS) personnel. Depending on the time of death, pre-hospital and hospital death can be differentiated. Most deaths occur in the pre-hospital setting. A study analysed all traumatic deaths in Berlin, where the main causes of death were polytrauma (45.7%), isolated severe traumatic brain injury (38%) and exsanguination (9.5%) [1]. 58.7% of these deaths occurred on site and 33.2% in the intensive care unit. Only 2.7% of deaths occurred in the emergency department (ED). Moreover, data on acute myocardial infarction shows that slightly more than 50% die in the pre-hospital setting, whereas about 20% die within the first 24 hours of being admitted to hospital [2].

The mortality rate in the ED is low, with a rate ranging from 0.2–0.3% [3,4]. However, our clinical impression is that some patients are being transported to hospital with no likelihood of survival, but demanding extensive hospital resources, such as resuscitation room infrastructure, and personnel from different disciplines, including intensive care, cardiology and trauma surgery. In the Franco-German EMS model, medical doctors often accompany the paramedics. This team provides care to the victims on site and on route and ensures rapid transport to the next appropriate hospital [5]. Dying patients are a special challenge for EMS personnel, as they require difficult decisions, such as whether to continue resuscitation when the chance of survival is uncertain. Decisions to intervene are always made without knowing the outcome. Furthermore, patient preferences should be respected by EMS.

Little is known about deaths at the ED. Epidemiological data are often non-existent. The term of “the golden hour” reflects the fact that early diagnosis and rapid treatment have a positive impact on the outcome [6,7]. To improve the chance that patients will survive transport, the first requirement is that all patients at risk need to be identified. Therefore, the purpose of this paper is to characterise this rather special population by describing the epidemiology of these patients, including pre-hospital and hospital information.

## Methods

### Study population

This retrospective cross-sectional study includes all adult patients transported by ground ambulance to the ED of a Swiss university hospital and who died during their stay in the ED. All causes of death—traumatic and due to illness—were included. Data were collected from January 2008 to December 2012. Only adult patients (>16 years) and primary transports by the main ambulance service *Sanitätspolizei* were included. We excluded patients who were transported by another EMS, by helicopter and self-referrals.

### Setting

The *Sanitätspolizei* is an EMS, which operates as a land-based rescue service for emergency care and ambulance transportation in the region of Berne. In 2012, the *Sanitätspolizei* operated a total of 16,827 transports. About three-quarters of these were primary transports. In recent years, the number of operations has increased. The triage of the EMS is based on the National Advisory Committee for Aeronautics (NACA) score, which measures the severity of an injury or an illness. This simple scoring system is mainly used in the pre-hospital setting and consists of 7 categories. NACA I stands for minor illness or trauma not requiring a medical intervention, whereas patients with NACA III suffer an illness or trauma requiring a hospital stay; NACA VI stands for resuscitation and NACA VII for death. [8].

The university hospital *Inselspital* provides 24-hour acute care with its medical and surgical ED. The ED has approximately 35,000 annual contacts and uses a four-level triage system, the Swiss Emergency Triage Scale (SETS). The SETS is a symptom-based triage scale: SETS level 1 represents life threatening situations that need immediate care, whereas SETS level 4 stand for non-urgent conditions [9].

### Data analysis

The study is based on the ambulance report forms of the EMS and on the discharge summaries of the ED. To guarantee data accuracy, cases were randomly checked to assure that they were clinically meaningful and coherent with other variables.

Epidemiological variables, such as cause of death, age and gender of all deceased patients were collected. Year, weekday and time of contact were retrieved. Pre-hospital information about the NACA score, operating time, duration of transportation, attendance of an emergency physician and initial therapeutic interventions were provided by the EMS. Hospital parameters such as further therapeutic interventions, electrocardiogram (ECG) findings and imaging were gathered from the ED. Therapeutic interventions comprised airway management (intubation, oxygen mask etc.), spine management (spine board, neck protector), defibrillation and pharmaceutical treatment. Vitals signs, such as systolic blood pressure (SBP), diastolic blood pressure (DBP), heart rate, peripheral oxygen saturation (SpO_2_) and the Glasgow Coma Scale (GCS) (first pre-hospital value and value on arrival) were collected. The Glasgow Coma Scale is an instrument to assess the level of consciousness, ranging from 3 points (coma) to 15 points (full level of consciousness) [10].

Information was collected on cardiopulmonary resuscitation (CPR) performed by professionals and CPR performed by bystanders. CPR was defined as chest compressions only (for bystanders) or together with artificial ventilation (for professionals). Since 2010, the *Sanitätspolizei* has been using a mechanical chest compression device (Lucas™2), and the use of this was also registered. Lucas™2 is a mechanical system that assists paramedics and doctors by providing uninterrupted chest compression. Therefore rescuers can focus on other life-saving tasks and transportation is facilitated. ([11], S1 and S2 Files).

The cause of death was based on the suspected diagnoses made by the physicians of the ED and was divided into traumatic deaths and deaths due to illness. Trauma stands for all accidents and suspected suicides by externally applied force.

The results are shown as numbers, percentages, medians and interquartile ranges (IQRs). Classical statistical tests were used for comparisons between groups (t test for parametric data; Mann-Whitney U test for non-parametric data).

### Ethics

Institutional review board approval was received from the *Swiss National Advisory Commission on Biomedical Ethics* (Approval No.21-02-13). The IRB waived consent from representatives. The hospital and ambulance data were linked and afterwards accessed and analysed anonymously.

## Results

### Study population

During the study period, the *Sanitätspolizei* transported 18,134 patients to the ED. 162 of these patients died in the ED. As one patient’s data was missing, a total of 161 patients were analysed in this study. 91% (n = 147) of the patients died due to diseases and 9% (n = 14) due to trauma. The median age was 70 years (IQR 56–81 years, age range 17–93 years) and 70% (n = 112) were men. Patients who died from trauma were significantly younger (median age 55 years, IQR 40–61 years, age range 18–83 years, p<0.001) and less often male (57%, n = 8, p = 0.280). The median pre-hospital NACA score was 6 (IQR 5–7) (Table 1).

**Table 1:** Epidemiological variables of the study population.

	Disease	Trauma	Total
**All Patients** n (%)	147 (91%)	14 (9%)	161 (100%)
**Sex** n (%)			
**Male**	104 (71%)	8 (57%)	112 (70%)
**Female**	43 (29%)	6 (43%)	49 (30%)
**Median Age** (IQR)	72 (59–82)	55 (40–61)	70 (56–81)
**Nationality** n (%)			
**Swiss**	136 (93%)	11 (79%)	147 (91%)
**Other Europeans**	8 (5%)	2 (14%)	10 (6%)
**Africans**	3 (2%)	0 (0%)	3 (2%)
**Asians**	0 (0%)	1 (7%)	1 (1%)
**NACA Score** ^a^ Median (IQR)	6 (5–7)	6 (5–6)	6 (5–7)

^a^ NACA score: National Advisory Committee for Aeronautics score

### Mortality and cause of death

The *Sanitätspolizei* transported 18,134 patients to the ED. The mortality was 0.9% (n = 161). The most common causes of death were cardiovascular (n = 70, 43%) and cerebrovascular (n = 14, 9%) events. Accidents and neoplasia were both responsible for 6% of deaths, followed by suspected suicide with 5% (n = 7). Deaths from abdominal (n = 3, 2%) or pulmonary causes (n = 3, 2%) were rare. One patient died from intoxication (1%). For some patients (27%, n = 44), the cause of death remained unclear. The most common cause of cardiovascular death was ischaemic heart disease (50%, n = 42), followed by pulmonary embolism (n = 20, 24%), aortic pathology (n = 13, 15%) and other heart diseases (n = 9, 11%). Most fatal injuries resulted from road traffic accidents (n = 6, 67%).

### Year, weekday and time of contact

**Fig 1:**
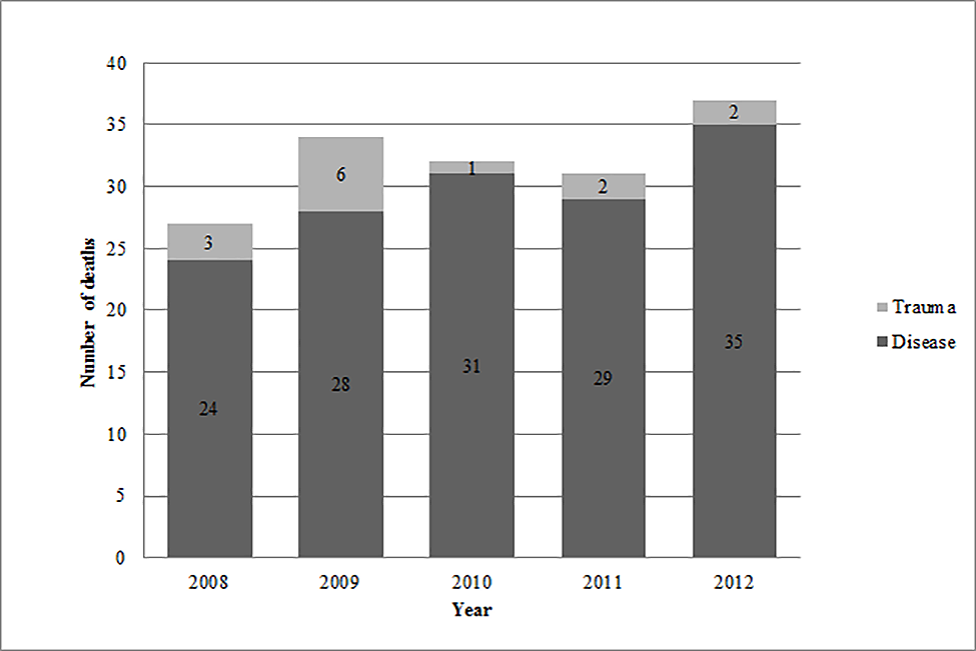
Annual numbers of deaths.

During 2008, 27 patients died in the ED, whereas in 2012, 37 patients died. (Fig 1) At the same time, primary transports to the ED increased. Transport was uniformly distributed over the week (Mon n = 26/16%, Tue n = 26/16%, Wed n = 24/15%, Tue n = 15/9%, Fri n = 22/14%, Sat n = 24/15%, Sun n = 24/15%). Most of the admissions of disease-related deaths were in the afternoon (n = 47, 32%), whereas traumatic deaths happened most at night (n = 5, 36%).

### Operating time and duration of transportation

The median time for the EMS to reach the operation site was 7 min (IQR 5–11 min). The operation site is the place where EMS pick up a patient. The median operating time at the site differed with the cause of death. Whereas for disease-related deaths it was 27 min (IQR 20–39 min), for traumatic deaths it was shorter with 20 min (IQR 16–29 min). The median time from the operation site to the ED was 10 min (IQR 6–14 min).

### Vital signs

The median pre-hospital heart rate was 90 bpm (IQR 70–105 bpm) and in 59% of patients (n = 95) the heart rate was not detectable. On arrival at the ED, the median heart rate was 92 bpm (IQR 80–100 bpm) and not detectable in 70% of patients (n = 113). In the pre-hospital setting, the median SBP and DBP were 118 mmHg (IQR 102–136 mmHg) and 80 mmHg (IQR 63–97 mmHg), respectively. In 63% of patients (n = 102), pre-hospital SBP und DBP were not detectable. On arrival at the ED, mean SBP and DBP decreased to 101 mmHg (IQR 80–140 mmHg) and 60 mmHg (IQR 40–77 mmHg), respectively. For 70% of patients (n = 112), SBP and DBP were not detectable at the ED.

The average pre-hospital SpO_2_ was 92% (IQR 83–97%), but was undetectable in 66% of patients (n = 107). In the ED, mean SpO_2_ increased to 97% (IQR 86–99%), but was undetectable in 75% of patients (n = 121). The median GCS, both pre-hospital and on arrival, was 3 (pre-hospital IQR 3–11, IQR at arrival 3–3).

In summary, the patients exhibited an increased heart rate and SpO_2_ on arrival. SBP and DBP were reduced on arrival. The number of undetectable vital signs increased on arrival. GCS stayed constantly low.

### Resuscitation

CPR by bystanders was performed in 34% (n = 50) of the disease-related deaths and in 21% (n = 3) of the traumatic deaths (p = 0.346). Professional CPR was conducted in 80% (n = 118) of the disease-related deaths and in 79% (n = 11) of the traumatic deaths. In 76% (n = 112) of the disease-related cases and in 100% (n = 14) of the traumatic cases, an emergency physician attended the operation and was part of the team (p = 0.037).

### Diagnostic interventions

#### ECG findings

92% of disease-related deaths (n = 135) and 71% (n = 10) of the trauma patients received an ECG. Asystole and pulseless electrical activity (PEA) were the most common ECG findings. Asystole was found in 35% (n = 51) of the disease-related deaths and in 43% (n = 6) of the traumatic deaths. PEA occurred in 58% (n = 85) of the disease-related deaths and in 43% (n = 6) of the traumatic deaths. Return of spontaneous circulation (ROSC) was observed infrequently (n = 13.8%). Disease-related deaths exhibited much greater variety in ECG findings.

#### Imaging

The most common imaging methods in disease-related deaths were echocardiography (n = 51, 35%), computed tomography (CT, n = 20, 14%) and FAST (n = 12, 8%), where FAST stands for focused assessment with sonography for trauma. For traumatic deaths, the most common imaging methods were FAST (n = 10, 71%), CT (n = 5, 36%) and Lodox (n = 4, 29%) Lodox is a low dose, full-body X-ray scanner.

### Therapeutic interventions

#### Pharmaceutical treatment

The most commonly used drugs were sympathomimetics/parasympathicolytics, narcotics/analgesics and volume replacement. The variety of medication was much lower in the pre-hospital setting and for traumatic deaths.

#### Airway management

**Fig 2:**
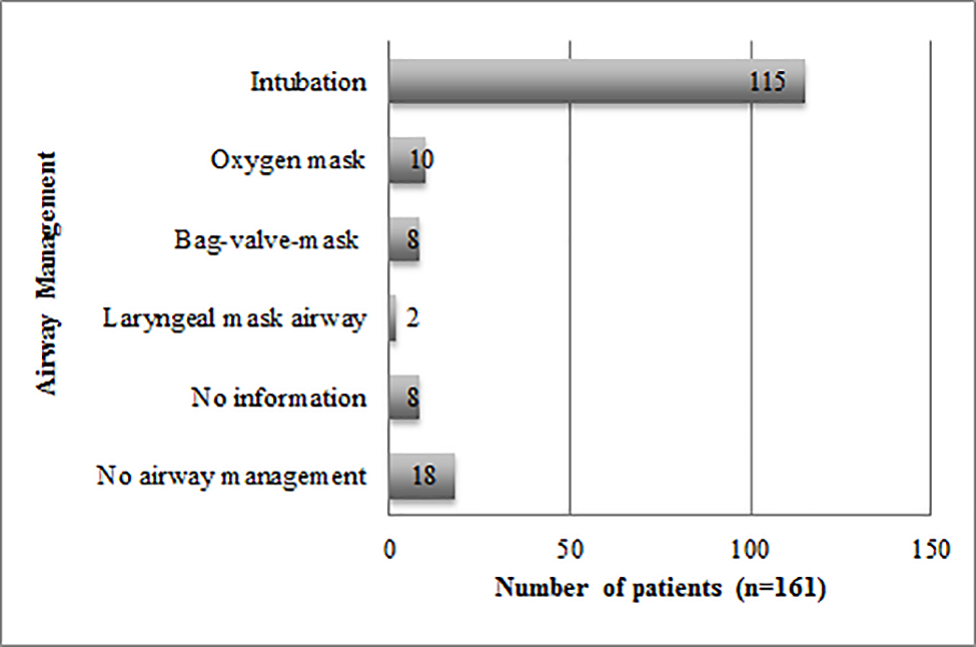
Airway management of all patients.

72% (n = 115) of all patients were intubated on site. Less frequently used airway devices included the oxygen face mask (n = 10, 6%), bag-valve-mask ventilation (n = 8, 5%) and the laryngeal mask airway (n = 2, 1%). 11% of patients (n = 18) received no airway management (Fig 2). 81% (n = 93) of the intubated patients showed no reflexes and were intubated without inducing anaesthesia. The variety of airway devices was much greater in the disease-related cases.

#### Spine management

Spine management included a spine board or stiff neck protector. Not all of the traumatic deaths received spine management (71%, n = 10). As expected, only a minority of the disease-related deaths received spine management (2%, n = 3).

#### Defibrillation

**Fig 3:**
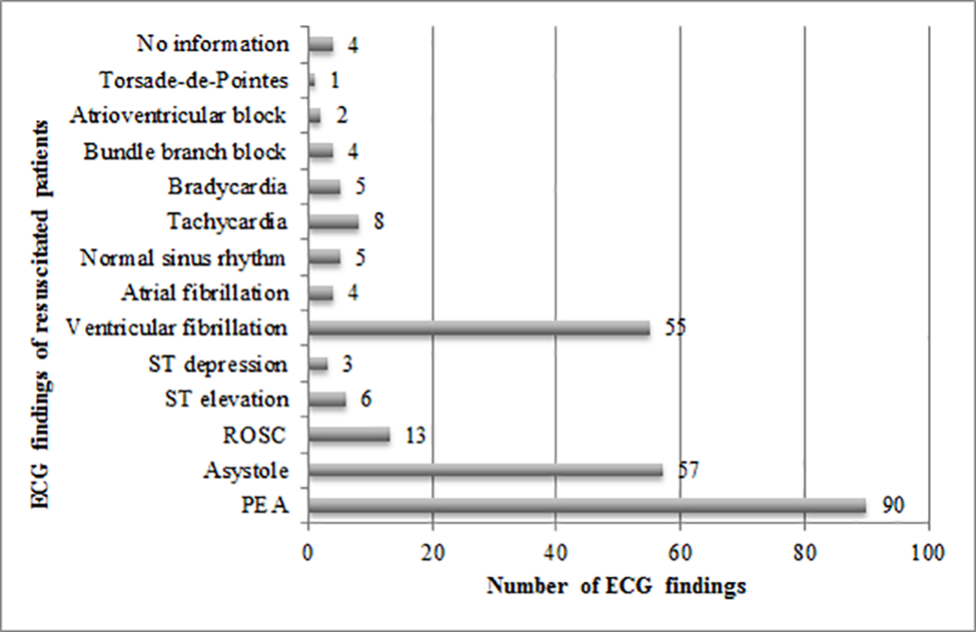
ECG findings of resuscitated patients.

43% (n = 63) of disease-related deaths received defibrillation. Only one patient with a traumatic death was defibrillated. Of the 129 patients (80%) who received professional CPR, half (n = 64, 50%) were defibrillated. For ECG findings of resuscitated patients, see Fig 3.

### Lucas™2

The automatic CPR device Lucas™2 has been used since 2010 and 43 patients in our study were resuscitated with this device. Of the patients with Lucas™ resuscitation, about half died from cardiovascular events (n = 21, 49%). For the other half (n = 21, 49%), the cause of death remained uncertain. Just one patient (2%) suffered trauma. With respect to the operating time on site, there was no relevant difference between use of Lucas™ (median 25 min, IQR 18–33 min) and no use of Lucas™ (median 26 min, IQR 20–35 min). Over 90% of the patients resuscitated with Lucas™ had no detectable vital signs. The most frequently ECG findings with Lucas™ resuscitation were PEA (n = 31), asystole (n = 26) and ventricular fibrillation (n = 19).

### Women versus men

**Fig 4:**
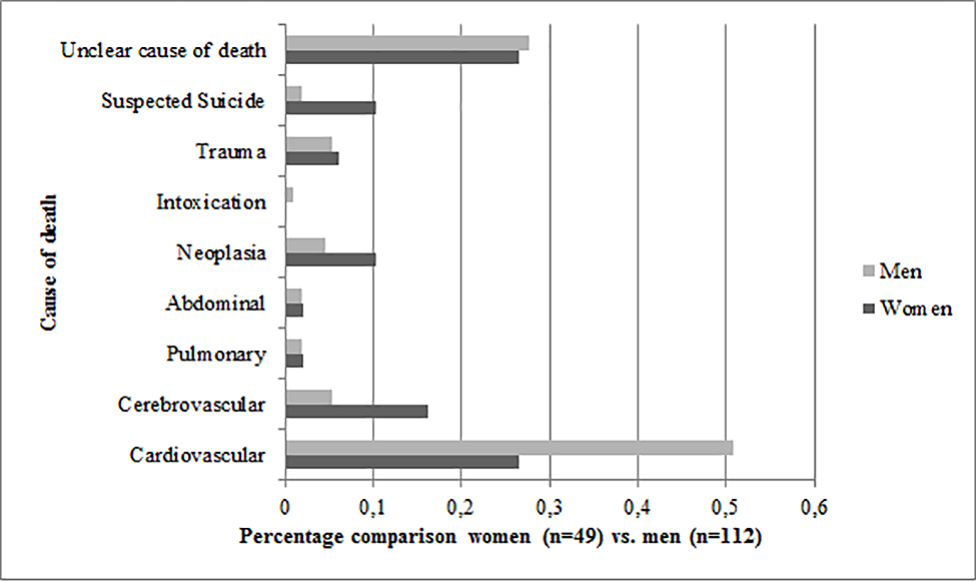
Percentage comparison women (n = 49) vs. men (n = 112) in cause of death.

More men (n = 112) than women (n = 49) died in the ED (p<0.001). There were also significant differences in the cause of death. Half of the causes of death in men were cardiovascular events (n = 57, 51%). For women, cardiovascular events made up only one quarter of the causes of death (n = 13, 27%, p<0.001). Cerebrovascular events (n = 8, 16%), neoplasia (n = 5, 10%) and suspected suicide (n = 5, 10%) were relatively more frequent for women (Fig 4). Men showed a higher rate of undetectable vital signs and professional resuscitation than women (n = 97, 87% vs. n = 32, 65%).

## Discussion

The purpose of this paper was to characterise this special population of patients dying in the ED. For all patients transported by EMS to the ED in this study, the mortality rate was 0.9%. The majority of deaths were due to cardiovascular diseases (43%). Trauma made up only 9%. The median age was 70 years and 70% were men. Trauma patients were younger (median age 55 years). Half of the men died due to cardiovascular events, whereas the causes of death in women varied more. One third of patients received CPR from bystanders, whereas professional CPR by the EMS was performed in most cases. Half of the resuscitated patients were defibrillated. The most common ECG findings were asystole and PEA. About three quarters of all patients were intubated in the pre-hospital setting. Our study did not find any difference in operating time on site when the mechanical chest compression device Lucas™ was employed.

This study has several limitations. The causes of death are based on discharge summaries of the ED and not on autopsy findings, which explains the high rate of unclear causes of death (27%). The study was conducted at one of the largest University Hospitals in Switzerland, and the only hospital in the catchment area offering third level medical and trauma care, together with the largest ambulance service in Switzerland. We therefore regard our sample as representative. Of course multicentre studies would give a larger sample size, but also with additional methodical obstacles, such as data clustering and additional technical and organisational workload. Patients transported by the helicopter emergency medical service (HEMS) to our ED have not been included in this study. If we had taken these patients into account, the distribution of causes of death might have changed (e.g. more trauma patients). Furthermore, a small number of data were missing, which seems to be unavoidable in the emergency setting.

One particular feature of this study is that it consists of pre-hospital data and data of the ED. This permits us to demonstrate the following trends in the vital signs: Heart rate and SpO_2_ increased on arrival at the ED, whereas systolic and diastolic BP decreased. Also the number of undetectable vital signs increased on arrival. The GCS remained low. These findings indicate that the patients’ condition continuously deteriorated.

The influence of pre-hospital time on trauma patients is often discussed in the literature. The systematic review of Harmsen et al. [12] showed that rapid transport was mainly beneficial for patients with unstable penetrating injuries. In our study, the operating time at the site for trauma patients was shorter than for disease-related patients.

A meta-analysis about predictors of out-of-hospital cardiac arrest proved that survival rate was higher when bystander CPR was performed or when the patient was found in shockable rhythm [13]. Nevertheless, the survival rate of cardiac arrest was very low (7.6%) [13], which was also demonstrated in our study.

Studies showed that ventricular fibrillation, ventricular tachycardia and ROSC were associated with a higher survival rate after cardiac arrest [13]. Those rhythms were less frequently detected in our population, which underlines these study findings.

It is notable that some of the trauma patients did not receive any spine management in the pre-hospital setting. These are individual cases and may be explained by special injury patterns (e.g. drowning, smoke inhalation/burning).

The rate of pre-hospital intubation is high in this study (72%), which might correlate with the low GCS and high number of emergency physicians. As in our findings, data from the German Trauma Register DGU show a high pre-hospital intubation rate for unconscious patients (90% when GCS≤8) [14]. Whether intubation should be conducted in cases of out-of-hospital cardiac arrest and whether it is better than other airway-devices is currently the subject of debate [15,16,17].

During the study period, the EMS established the use of Lucas™2. Recent trials [18,19,20] demonstrated that mechanical compression devices did not improve the survival rates of out-of-hospital cardiac arrest in comparison to manual CPR. However, the use of Lucas™2 seems reasonable when high-quality manual CPR cannot be guaranteed due to difficult circumstances [21]. Our study did not show any difference in operating time on site when Lucas™ was used, but it seems to be a feasible and comfortable tool for EMS personnel.

### Implications

It has been suggested that EMS personnel transports too many patients to the ED who are already dead or in the early stages of death. The low overall ED mortality rate in this study contradicts this subjective impression. For this study population, therapeutic measures have not been successful and one can discuss whether interventions were futile and whether resuscitation should have been terminated in the pre-hospital setting. Further investigations and studies would be needed to establish the lack of causality (or futility) of specific interventions.

The high average age of 70 years indicates that age plays a role, especially in patients with medical conditions. One can discuss whether age should be considered more in the pre-hospital triage process. In some newer triage scoring systems, age is a fixed component (e.g. the *Mechanism*, *GCS*, *Age and Pressure (MGAP) score* for trauma [22]).

The rate of CPR by bystanders was low in this study. This observation might be due to several different causes, including emotional shock, lack of knowledge and skills and lack of self-confidence [23]. As early CPR correlates with a higher survival rate [13], it is crucial that public awareness and training of CPR are promoted further.

## Conclusion

The mortality rate in this study was low with 0.9% and contradicts the impression that EMS personnel transport too many patients with no chance of survival to the ED. The main cause of death was of cardiovascular, with a high median age of 70 years. Taking the ECG findings and vital signs into account, the majority of these patients were in a precarious state of health. CPR by bystanders was conducted in only one third of the patients, which demonstrates that public CPR training could be promoted further. The use of Lucas™2 might be useful, but did not reduce the operating time on site.

Characterisation of these patients is a first step to creating a better understanding of transporting the dying patient in an urban setting. As a cross-sectional study, this paper gives an epidemiological overview. Comparison with survivors might provide further insights, possibly including the futility of individual interventions.

## Supporting information

S1 File Minimal data set. (PDF)

S2 File Legend minimal data set. (PDF)
