# Supplementary material for: The dead and the dying - a difficult part of EMS transport: A Swiss cross-sectional study
Source: PLoS One. 2018 Feb 27;13(2):e0191879. doi: 10.1371/journal.pone.0191879 (PMC5828436; doi:10.1371/journal.pone.0191879)
Supplement: S1 File — (PDF) [file pone.0191879.s001.pdf]

Patient numb	Year	Time of start	Arrival operati	Departure ope	Arrival ED	Time to opera
1	2008	02:50	02:58	03:59	04:05	00:08
2	2008	13:56	14:03	14:25	14:30	00:06
3	2008	15:25	15:55	16:15	16:32	00:30
4	2008	20:13	20:18	20:45	20:50	00:05
5	2008	19:24	19:26	19:35	19:40	00:02
6	2008	12:33	12:45	13:25	13:35	00:12
7	2008	14:08	14:15	14:30	14:35	00:07
8	2008	08:40	08:44	08:57	09:11	00:04
9	2008	01:48	01:56	02:20	02:30	00:08
10	2008	19:53	20:10	20:17	20:30	00:17
11	2008	17:32	17:36	17:50	17:55	00:04
12	2008	11:18	11:32	11:55	12:14	00:14
13	2008	23:20	23:28	00:13	00:18	00:08
14	2008	18:24	18:35	19:00	19:15	00:11
15	2008	17:24	17:35	18:17	18:35	00:11
16	2008	18:44	18:58	19:10	19:27	00:14
17	2008	20:23	20:30	21:00	21:10	00:07
18	2008	14:38	14:43	15:18	15:30	00:05
19	2008	19:23	19:27	19:40	19:47	00:04
20	2008	00:12	00:25	01:43	01:55	00:13
21	2008	09:26	09:30	10:10	10:20	00:04
22	2008	19:09	19:15	19:40	19:48	00:06
23	2008	01:17	01:28	02:00	02:15	00:11
24	2008	02:27	02:37	03:07	03:22	00:10
25	2008	01:59	02:25	03:45	03:49	00:26
26	2008	12:38	12:43	13:25	13:30	00:05
27	2008	14:57	15:02	15:50	16:00	00:05
28	2009	22:45	22:50	23:10	23:25	00:05
29	2009	04:57	05:13	05:36	05:50	00:16
30	2009	08:43	08:54	09:20	09:29	00:11
31	2009	02:09	02:15	03:25	03:35	00:06
32	2009	21:05	21:25	21:30	21:40	00:20
33	2009	18:33	18:40	19:55	20:10	00:07
34	2009	13:47	13:57	14:25	14:35	00:10
35	2009	12:35	12:42	12:55	13:05	00:07
36	2009	16:21	16:23	16:44	16:47	00:02
37	2009	16:49	16:50	17:10	17:20	00:01
38	2009	00:44	00:54	01:20	01:35	00:10
39	2009	01:47	01:53	02:06	02:16	00:06
40	2009	04:24	04:30	05:45	05:55	00:06
41	2009	10:32	10:45	11:26	11:41	00:13
42	2009	10:41	10:53	11:39	11:44	00:12
43	2009	15:37	15:43	16:00	16:01	00:06

44	2009	00:16	00:23	01:06	01:10	00:07
45	2009	01:42	01:50	02:20	02:25	00:08
46	2009	08:55	09:07	09:46	10:02	00:12
47	2009	08:23	08:35	09:15	09:33	00:12
48	2009	18:11	18:20	18:30	18:40	00:09
49	2009	23:49	23:55	01:08	01:18	00:06
50	2009	12:48	13:03	13:23	13:40	00:15
51	2009	05:00	05:12	05:38	05:52	00:12
52	2009	21:39	21:45	22:30	22:40	00:06
53	2009	23:09	23:14	23:50	23:57	00:05
54	2009	19:12	19:20	19:40	19:45	00:08
55	2009	16:11	16:25	16:45	17:00	00:14
56	2009	17:47	17:55	18:18	18:27	00:08
57	2009	01:19	01:30	02:00	02:15	00:11
58	2009	20:13	20:16	20:50	20:55	00:03
59	2009	11:41	11:46	12:10	12:15	00:05
60	2009	11:18	11:26	11:37	11:44	00:08
61	2009	21:08	21:10	21:30	21:40	00:02
62	2010	09:50	10:02	10:27	10:40	00:12
63	2010	22:01	22:03	22:50	22:55	00:02
64	2010	08:31	08:38	09:24	09:39	00:07
65	2010	19:15	19:30	19:55	20:05	00:15
66	2010	07:24	07:27	08:02	08:07	00:03
67	2010	08:44	08:47	08:59	09:02	00:03
68	2010	20:23	21:00	21:25	21:26	00:37
69	2010	14:45	14:54	15:27	15:31	00:09
70	2010	15:23	15:35	15:45	16:00	00:12
71	2010	10:37	10:44	11:05	11:15	00:07
72	2010	07:16	07:20	07:56	08:04	00:04
73	2010	03:25	03:35	04:17	04:32	00:10
74	2010	16:48	16:53	17:36	17:43	00:05
75	2010	08:22	08:27	09:00	09:07	00:05
76	2010	21:30	21:45	22:05	22:20	00:15
77	2010	11:46	11:55	12:20	12:30	00:09
78	2010	21:06	21:15	21:45	22:00	00:09
79	2010	12:02	12:11	12:34	12:38	00:09
80	2010	15:57	16:00	16:20	16:25	00:03
81	2010	04:12	04:18	04:48	05:08	00:06
82	2010	21:52	21:54	22:30	22:33	00:02
83	2010	18:44	18:53	19:15	19:20	00:09
84	2010	15:28	15:40	16:10	16:16	00:12
85	2010	09:47	09:55	10:25	10:40	00:08
86	2010	17:34	17:38	18:30	18:35	00:04
87	2010	12:51	12:55	13:53	14:00	00:04

88	2010	17:17	17:30	17:50	18:10	00:13
89	2010	14:41	14:53	15:20	15:30	00:12
90	2010	14:00	14:09	14:31	14:48	00:09
91	2010	09:41	09:44	09:54	10:04	00:03
92	2010	13:25	13:32	14:05	14:20	00:07
93	2010	18:27	18:32	18:55	19:02	00:05
94	2011	11:00	11:15	11:47	11:56	00:15
95	2011	09:44	09:52	10:16	10:25	00:08
96	2011	DM	DM	DM	DM	DM
97	2011	14:28	14:38	14:58	15:05	00:10
98	2011	06:00	06:12	06:32	06:39	00:12
99	2011	01:25	01:31	01:56	02:05	00:06
100	2011	17:37	17:38	18:00	18:04	00:01
101	2011	09:59	10:04	10:31	10:34	00:05
102	2011	23:35	23:50	00:05	00:33	00:15
103	2011	12:13	12:20	12:34	12:37	00:07
104	2011	10:54	11:04	11:15	11:25	00:10
105	2011	16:44	16:55	17:07	17:25	00:11
106	2011	19:20	19:23	19:50	19:58	00:03
107	2011	12:38	12:46	13:13	13:18	00:08
108	2011	01:22	01:28	01:42	01:55	00:06
109	2011	23:55	23:59	00:40	00:47	00:04
110	2011	14:07	14:15	14:35	14:45	00:08
111	2011	16:38	16:41	16:55	17:01	00:03
112	2011	15:16	15:27	16:06	16:25	00:11
113	2011	13:24	DM	DM	DM	DM
114	2011	21:17	21:28	21:57	22:13	00:11
115	2011	10:55	10:58	11:24	11:27	00:03
116	2011	10:05	10:12	10:45	10:52	00:07
117	2011	14:39	14:45	15:15	15:20	00:06
118	2011	12:11	12:12	12:52	12:55	00:01
119	2011	15:22	15:35	15:47	15:57	00:13
120	2011	09:16	09:26	09:55	10:06	00:10
121	2011	10:18	10:26	11:10	11:26	00:08
122	2011	17:15	17:18	17:26	17:59	00:03
123	2011	11:50	12:00	12:10	12:20	00:10
124	2011	15:38	15:50	16:25	16:45	00:12
125	2012	10:26	10:33	10:48	10:56	00:07
126	2012	13:01	13:07	13:50	13:55	00:06
127	2012	10:00	10:04	10:37	10:45	00:04
128	2012	10:07	10:15	10:35	10:45	00:08
129	2012	12:48	12:53	13:15	13:19	00:05
130	2012	16:52	16:55	17:14	17:19	00:03
131	2012	16:54	16:58	17:50	17:56	00:04

132	2012	14:33	14:45	15:00	15:11	00:12
133	2012	16:34	16:38	17:10	17:18	00:04
134	2012	13:46	13:50	14:15	14:22	00:04
135	2012	12:25	12:30	12:40	12:45	00:05
136	2012	01:42	01:46	02:10	02:22	00:04
137	2012	09:20	09:29	10:09	10:14	00:09
138	2012	22:33	22:37	23:17	23:28	00:04
139	2012	12:29	12:35	13:08	13:15	00:06
140	2012	13:49	14:00	14:40	15:00	00:11
141	2012	22:07	22:10	22:35	22:50	00:03
142	2012	18:08	18:15	18:35	18:41	00:07
143	2012	15:02	15:09	15:40	15:50	00:07
144	2012	11:30	11:40	12:23	12:35	00:10
145	2012	11:05	11:15	11:50	12:00	00:10
146	2012	19:34	19:38	19:56	20:03	00:04
147	2012	06:56	07:01	08:02	08:10	00:05
148	2012	12:42	12:48	13:08	13:15	00:06
149	2012	11:21	11:26	11:55	12:04	00:05
150	2012	17:34	17:40	18:45	18:55	00:06
151	2012	11:07	11:10	11:25	11:30	00:03
152	2012	09:29	09:32	10:13	10:18	00:03
153	2012	07:19	07:25	07:50	07:57	00:06
154	2012	21:02	21:06	21:35	21:46	00:04
155	2012	17:38	17:46	17:55	18:04	00:08
156	2012	14:23	14:35	14:50	15:20	00:12
157	2012	14:44	14:58	15:13	15:30	00:14
158	2012	23:33	23:40	01:00	01:10	00:07
159	2012	03:58	04:00	04:17	04:25	00:02
160	2012	16:25	16:29	17:04	17:10	00:04
161	2012	07:44	07:48	08:04	08:11	00:04

Time at operat	Time to hospit	NACA Score	Heart rate pre	SBP pre-hospit	DBP pre-hospi	SpO2 pre-hosp
01:01	00:06	6	105	90	60	87
00:22	00:05	DM	90	100	60	9999
00:20	00:17	6	88	180	157	83
00:27	00:05	7	9999	9999	9999	9999
00:09	00:05	5	65	150	110	84
00:40	00:10	6	104	78	38	87
00:15	00:05	7	9999	9999	9999	9999
00:13	00:14	3	79	113	84	80
00:24	00:10	5	98	98	56	83
00:07	00:13	3	60	170	110	99
00:14	00:05	7	9999	9999	9999	9999
00:23	00:19	6	38	135	85	84
00:45	00:05	7	9999	9999	9999	9999
00:25	00:15	7	135	105	68	85
00:42	00:18	7	42	9999	9999	9999
00:12	00:17	3	140	100	55	84
00:30	00:10	7	9999	9999	9999	9999
00:35	00:12	6	9999	9999	9999	9999
00:13	00:07	7	9999	9999	9999	9999
01:18	00:12	6	9999	9999	9999	9999
00:40	00:10	5	92	109	82	86
00:25	00:08	6	9999	9999	9999	9999
00:32	00:15	4	82	91	73	93
00:30	00:15	5	75	9999	9999	99
01:20	00:04	6	9999	9999	9999	9999
00:42	00:05	6	9999	9999	9999	9999
00:48	00:10	6	11	125	65	69
00:20	00:15	6	9999	9999	9999	9999
00:23	00:14	5	80	160	67	100
00:26	00:09	4	42	103	85	92
01:10	00:10	6	9999	9999	9999	9999
00:05	00:10	3	95	120	75	98
01:15	00:15	6	56	9999	9999	97
00:28	00:10	7	9999	9999	9999	9999
00:13	00:10	7	9999	9999	9999	9999
00:21	00:03	7	9999	9999	9999	9999
00:20	00:10	5	125	118	95	9999
00:26	00:15	5	65	80	60	94
00:13	00:10	6	9999	9999	9999	9999
01:15	00:10	6	40	150	115	9999
00:41	00:15	6	9999	9999	9999	9999
00:46	00:05	5	120	125	95	9999
00:17	00:01	7	9999	9999	9999	9999

00:43	00:04	6	9999	9999	9999	9999
00:30	00:05	4	85	220	130	93
00:39	00:16	6	9999	9999	9999	9999
00:40	00:18	5	65	99	65	96
00:10	00:10	7	9999	9999	9999	9999
01:13	00:10	7	9999	9999	9999	9999
00:20	00:17	7	45	115	75	90
00:26	00:14	7	140	9999	9999	9999
00:45	00:10	7	9999	9999	9999	9999
00:36	00:07	6	104	9999	9999	9999
00:20	00:05	5	97	204	109	100
00:20	00:15	6	9999	9999	9999	9999
00:23	00:09	6	9999	9999	9999	9999
00:30	00:15	7	9999	9999	9999	9999
00:34	00:05	7	9999	9999	9999	9999
00:24	00:05	7	9999	9999	9999	9999
00:11	00:07	6	9999	9999	9999	9999
00:20	00:10	7	9999	9999	9999	9999
00:25	00:13	7	9999	9999	9999	9999
00:47	00:05	6	9999	9999	9999	9999
00:46	00:15	7	47	50	30	81
00:25	00:10	6	9999	9999	9999	9999
00:35	00:05	5	48	222	80	99
00:12	00:03	7	9999	9999	9999	9999
00:25	00:01	5	95	175	95	92
00:33	00:04	6	32	130	55	9999
00:10	00:15	4	90	110	80	98
00:21	00:10	5	95	105	85	100
00:36	00:08	7	9999	9999	9999	9999
00:42	00:15	6	9999	9999	9999	9999
00:43	00:07	6	9999	9999	9999	9999
00:33	00:07 DM		84	136	109	92
00:20	00:15	6	9999	9999	9999	9999
00:25	00:10	7	9999	9999	9999	9999
00:30	00:15	7	9999	9999	9999	9999
00:23	00:04	7	9999	9999	9999	9999
00:20	00:05	7	9999	9999	9999	9999
00:30	00:20	5	85	185	100	99
00:36	00:03	6	9999	9999	9999	9999
00:22	00:05	5	83	103	72	84
00:30	00:06	6	9999	9999	9999	9999
00:30	00:15	7	9999	9999	9999	9999
00:52	00:05	7	9999	9999	9999	9999
00:58	00:07	6	9999	9999	9999	9999

00:20	00:20	3	95	125	72	9999
00:27	00:10	6	9999	9999	9999	9999
00:22	00:17	7	9999	9999	9999	9999
00:10	00:10	7	130	115	65	68
00:33	00:15	7	9999	9999	9999	9999
00:23	00:07	6	9999	9999	9999	9999
00:32	00:09	4	102	125	105	77
00:24	00:09	7	9999	9999	9999	9999
DM	DM	4	120	DM	DM	90
00:20	00:07	6	9999	9999	9999	9999
00:20	00:07	5	95	115	80	9999
00:25	00:09	6	44	9999	9999	68
00:22	00:04	6	9999	9999	9999	9999
00:27	00:03	6	9999	9999	9999	9999
00:15	00:28	7	110	75	50	80
00:14	00:03	3	DM	DM	DM	DM
00:11	00:10	7	9999	9999	9999	9999
00:12	00:18	5	90	130	70	98
00:27	00:08	6	9999	9999	9999	9999
00:27	00:05	5	58	120	103	50
00:14	00:13	3	72	130	85	91
00:41	00:07	5	110	105	60	97
00:20	00:10	3	75	140	80	97
00:14	00:06	5	125	204	120	97
00:39	00:19	7	9999	9999	9999	9999
DM	DM	5	110	110	80	84
00:29	00:16	6	9999	9999	9999	9999
00:26	00:03	5	93	77	40	97
00:33	00:07	7	9999	9999	9999	9999
00:30	00:05	7	9999	9999	9999	9999
00:40	00:03	6	9999	9999	9999	9999
00:12	00:10	7	9999	9999	9999	9999
00:29	00:11	7	9999	9999	9999	9999
00:44	00:16	6	99	90	60	60
00:08	00:33	7	9999	9999	9999	9999
00:10	00:10	5	9999	9999	9999	9999
00:35	00:20	5	89	115	40	95
00:15	00:08	6	9999	9999	9999	9999
00:43	00:05	7	9999	9999	9999	9999
00:33	00:08	6	9999	9999	9999	9999
00:20	00:10	6	70	90	70	94
00:22	00:04	6	9999	9999	9999	9999
00:19	00:05	7	9999	9999	9999	9999
00:52	00:06	6	9999	9999	9999	9999

00:15	00:11	7	9999	9999	9999	9999
00:32	00:08	6	9999	9999	9999	9999
00:25	00:07	7	9999	9999	9999	9999
00:10	00:05	5	90	120	80	98
00:24	00:12	4	115	120	98	89
00:40	00:05	7	130	133	100	DM
00:40	00:11	6	9999	9999	9999	9999
00:33	00:07	5	119	131	83	70
00:40	00:20	6	9999	9999	9999	9999
00:25	00:15	7	9999	9999	9999	9999
00:20	00:06	7	73	147	126	73
00:31	00:10	6	124	108	65	80
00:43	00:12	7	9999	9999	9999	9999
00:35	00:10	4	95	85	55	93
00:18	00:07	6	9999	9999	9999	9999
01:01	00:08	7	9999	9999	9999	9999
00:20	00:07	7	9999	9999	9999	9999
00:29	00:09	7	9999	9999	9999	9999
01:05	00:10	6	9999	9999	9999	9999
00:15	00:05	7	9999	9999	9999	9999
00:41	00:05	6	9999	9999	9999	9999
00:25	00:07	7	9999	9999	9999	9999
00:29	00:11	7	9999	9999	9999	9999
00:09	00:09	7	9999	9999	9999	9999
00:15	00:30	7	9999	9999	9999	9999
00:15	00:17	7	9999	9999	9999	9999
01:20	00:10	3	70	95	60	9999
00:17	00:08	5	115	150	100	98
00:35	00:06	7	9999	9999	9999	9999
00:16	00:07	6	9999	9999	9999	9999

GCS pre-hospi	Heart rate ED	SBD ED (mmH	DBD ED (mmH	SpO2 ED (%)	GCS ED	Attendance er
3	55	80	40	87	3	yes
3	9999	9999	9999	9999	3	yes
15	9999	9999	9999	9999	3	yes
3	9999	9999	9999	9999	3	yes
14	80	40	20	70	DM	yes
7	9999	9999	9999	9999	3	yes
3	9999	9999	9999	9999	3	yes
15	135	154	94	69	DM	no
15	88	115	68	100	DM	yes
15	40	166	108	99	DM	no
3	9999	9999	9999	9999	3	yes
3	9999	9999	9999	9999	3	yes
3	9999	9999	9999	9999	3	yes
14	9999	9999	9999	9999	3	yes
3	9999	9999	9999	9999	3	yes
11	133	195	46	93	DM	no
3	9999	9999	9999	9999	3	yes
3	9999	9999	9999	9999	3	yes
3	9999	9999	9999	9999	3	yes
3	9999	9999	9999	9999	3	yes
14	100	100	70	100	3	yes
3	9999	9999	9999	9999	3	yes
14	97	61	40	84	DM	no
14	90	90	50	100	DM	yes
3	9999	9999	9999	9999	3	yes
3	9999	9999	9999	9999	3	yes
3	9999	9999	9999	9999	3	yes
3	9999	9999	9999	9999	3	yes
10	110	189	93	87	3	no
14	9999	9999	9999	88	3	no
3	120	54	38	98	3	yes
15	109	184	74	95	DM	no
15	9999	9999	9999	9999	3	yes
3	9999	9999	9999	9999	3	yes
3	9999	9999	9999	9999	3	yes
3	9999	9999	9999	9999	3	yes
3	40	65	40	93	3	yes
15	90	85	65	79	14	yes
3	9999	9999	9999	9999	3	yes
3	9999	9999	9999	9999	3	yes
3	9999	9999	9999	9999	3	yes
11	9999	9999	9999	9999	3	no
3	9999	9999	9999	9999	3	yes

3	110	110	45	9999	3	yes
15	DM	260	120	DM	3	yes
3	120	53	37	9999	3	yes
15	140	100	64	9999	DM	yes
3	9999	9999	9999	9999	3	yes
3	9999	9999	9999	9999	3	yes
13	9999	9999	9999	9999	3	yes
3	9999	9999	9999	9999	3	no
3	9999	9999	9999	9999	3	yes
3	9999	9999	9999	83	3	yes
6	97	142	74	94	5	yes
3	9999	9999	9999	9999	3	yes
3	9999	9999	9999	9999	3	yes
3	9999	9999	9999	9999	3	yes
3	9999	9999	9999	9999	3	yes
3	9999	9999	9999	9999	3	yes
3	9999	9999	9999	9999	3	yes
3	9999	9999	9999	9999	3	yes
3	9999	9999	9999	9999	3	yes
3	9999	9999	9999	9999	3	yes
5	9999	9999	9999	9999	3	yes
3	9999	9999	9999	9999	3	yes
3	96	238	68	99	3	yes
3	9999	9999	9999	9999	3	yes
6	84	196	105	99	3	yes
3	9999	9999	9999	9999	3	yes
14	80	42	25	DM	DM	yes
14	75	90	50	94	10	yes
3	9999	9999	9999	9999	3	yes
3	9999	9999	9999	9999	3	yes
3	9999	9999	9999	9999	3	yes
9	24	80	60	DM	3	yes
3	9999	9999	9999	9999	3	no
3	9999	9999	9999	9999	3	yes
3	9999	9999	9999	9999	3	yes
3	9999	9999	9999	9999	3	yes
3	9999	9999	9999	9999	3	yes
3	100	104	54	99	3	yes
3	9999	9999	9999	9999	3	yes
10	87	155	95	99	9	yes
3	86	150	90	DM	3	yes
3	9999	9999	9999	9999	3	yes
3	9999	9999	9999	9999	3	yes
3	60	140	121	96	3	yes

	14	103	128	84	75	13	no
	3	9999	9999	9999	9999	3	yes
	3	9999	9999	9999	9999	3	yes
	15	9999	9999	9999	9999	3	yes
	3	9999	9999	9999	9999	3	yes
	3	9999	9999	9999	9999	3	yes
	15	9999	9999	9999	9999	6	no
	3	9999	9999	9999	9999	3	yes
DM		81	55	28	81	13	no
	3	9999	9999	9999	9999	3	yes
	14	100	80	40	9999	14	yes
	3	92	70	38	99	3	yes
	3	9999	9999	9999	9999	3	yes
	3	9999	9999	9999	9999	3	yes
	15	9999	9999	9999	9999	3	no
	13	DM	DM	DM	DM	DM	no
	3	9999	9999	9999	9999	3	no
	5	90	120	70	99	5	yes
	3	9999	9999	9999	9999	3	no
	11	92	98	78	9999	4	no
	15	79	112	77	99	15	yes
	3	110	89	71	99	3	yes
	15	71	197	55	100	15	no
	5	120	133	70	100	3	no
	3	9999	9999	9999	9999	3	yes
	15	9999	9999	9999	9999	3	no
	3	9999	9999	9999	9999	3	yes
	15	130	9999	9999	9999	15	no
	3	9999	9999	9999	9999	3	yes
	3	9999	9999	9999	9999	3	yes
	3	9999	9999	9999	9999	3	no
	3	9999	9999	9999	9999	3	no
	3	9999	9999	9999	9999	3	yes
	15	9999	9999	9999	9999	3	yes
	3	9999	9999	9999	9999	3	no
	6	125	110	80	88	3	yes
	6	68	60	40	100	3	yes
	3	9999	9999	9999	9999	3	no
	3	9999	9999	9999	9999	3	no
	3	9999	9999	9999	9999	3	yes
	3	80	55	20	DM	3	no
	3	63	102	55	99	3	yes
	3	9999	9999	9999	9999	3	yes
	3	DM	DM	DM	DM	3	yes

3	9999	9999	9999	9999	3	yes
3	9999	9999	9999	9999	3	yes
3	9999	9999	9999	9999	3	no
15	93	101	73	98	15	yes
15	120	80	48	82	15	no
15	9999	9999	9999	9999	3	no
15	9999	9999	9999	9999	3	yes
14	99	126	99	81	6	yes
3	80	50	10	75	3	yes
3	9999	9999	9999	9999	3	yes
3	9999	9999	9999	9999	3	no
15	9999	9999	9999	9999	3	yes
3	9999	9999	9999	9999	3	yes
15	89	140	60	100	15	no
3	9999	9999	9999	9999	3	yes
3	9999	9999	9999	9999	3	yes
3	9999	9999	9999	9999	3	yes
3	9999	9999	9999	9999	3	yes
3	9999	9999	9999	9999	3	yes
3	9999	9999	9999	9999	3	yes
3	9999	60	30	9999	3	yes
3	9999	9999	9999	9999	3	no
3	9999	9999	9999	9999	3	yes
15	9999	9999	9999	9999	3	yes
6	9999	9999	9999	9999	3	yes
3	9999	9999	9999	9999	3	no
14	DM	DM	DM	DM	DM	yes
3	117	92	43	99	3	yes
3	9999	9999	9999	9999	3	yes
3	9999	9999	9999	9999	3	yes

CPR by bystan	Professional C	Airway manag	Method of int	Spine Manage	Pharmaceutca	Pharmaceuti
no	yes	intubation	no reflexes	no	Adrenaline, A	Adrenaline, A
yes	yes	intubation	anaesthesia	no	Adrenaline, E	-
no	yes	intubation	DM	no	Aziclav	unspecified C
no	yes	intubation	no reflexes	no	Adrenaline, A	-
no	no	intubation	anaesthesia	no	Etomidate, Fe	Adrenaline
no	yes	intubation	anaesthesia	no	Etomidate, Fe	Adrenaline, A
no	yes	intubation	no reflexes	no	Adrenaline	-
no	no	oxygen face r	-	no	-	Lasix, Cepime
no	no	no	-	no	Isoket Spray,	Plavix, Liquen
no	no	no	-	no	Methylpredn	Rivotril, Solur
yes	yes	intubation	no reflexes	no	Adrenaline	-
no	yes	intubation	no reflexes	no	Adrenaline	Adrenaline
yes	yes	intubation	no reflexes	no	Atropine, Adr	unspecified C
no	yes	intubation	no reflexes	no	Atropine, Adr	Adrenaline, A
yes	yes	DM	-	no	Atropine, Adr	-
no	no	oxygen face r	-	no	-	Beloc, Lasix, M
no	yes	intubation	no reflexes	no	Adrenaline	-
yes	yes	intubation	anaesthesia	no	Adrenaline, C	-
yes	yes	intubation	no reflexes	no	Adrenaline, A	-
no	yes	intubation	no reflexes	no	Adrenaline, A	-
no	no	intubation	DM	no	-	-
yes	yes	intubation	no reflexes	yes	Adrenaline, A	-
no	yes	oxygen face r	-	no	Ephedrin, Na	Adrenaline, A
no	no	oxygen face r	-	no	-	Morphine
no	yes	intubation	no reflexes	no	Adrenaline, A	-
no	yes	intubation	anaesthesia	no	Adrenaline, A	-
no	yes	intubation	no reflexes	yes	Adrenaline, A	-
yes	yes	intubation	anaesthesia (	no	Adrenaline, A	Cordaron, Ad
no	no	intubation	DM	no	Torecan	-
no	yes	intubation	DM	no	-	unspecified C
no	yes	intubation	no reflexes	no	Adrenaline, A	Adrenaline, N
no	no	oxygen face r	-	no	-	-
no	yes	intubation	anaesthesia	no	Adrenaline, A	-
yes	yes	intubation	no reflexes	no	Adrenaline, C	-
yes	yes	no	-	no	Adrenaline, A	-
no	yes	intubation	no reflexes	no	Adrenaline, A	-
no	yes	intubation	anaesthesia	yes	Adrenaline, N	Adrenaline
no	no	no	-	no	Adrenaline, E	-
no	yes	intubation	no reflexes	yes	Adrenaline	Catecholamir
no	yes	intubation	no reflexes	no	Adrenaline, V	unspecified C
no	yes	intubation	no reflexes	no	Adrenaline, A	-
no	yes	intubation	no reflexes	yes	-	Adrenaline, A
no	yes	intubation	no reflexes	no	Magnesium, I	-

no	yes	intubation	no reflexes	no	Adrenaline, A	Sandostatin, I
no	no	no	-	no	-	-
no	yes	intubation	no reflexes	no	Adrenaline, A	Adrenaline, A
no	yes	oxygen face r	-	no	Nitro, Aspegi	Adrenaline, C
yes	yes	bag-valve-ma	-	no	Adrenaline, A	Adrenaline
yes	yes	intubation	no reflexes	no	Adrenaline, A	-
no	yes	bag-valve-ma	-	no	Adrenaline	Adrenaline
no	yes	intubation	no reflexes	no	Adrenaline, A	-
yes	yes	intubation	no reflexes	no	Adrenaline, A	Adrenaline, A
no	yes	intubation	no reflexes	yes	Adrenaline, A	Adrenaline, A
no	no	intubation	DM	no	-	-
no	yes	intubation	no reflexes	no	Adrenaline, A	Adrenaline, N
yes	yes	intubation	no reflexes	no	Phenylephrin	Adrenaline
no	yes	intubation	no reflexes	no	Adrenaline, A	-
yes	yes	intubation	no reflexes	yes	Adrenaline, A	-
yes	yes	intubation	no reflexes	no	Adrenaline, A	-
yes	yes	intubation	no reflexes	no	Adrenaline, A	Adrenaline, E
no	yes	intubation	no reflexes	no	Adrenaline, C	-
no	yes	intubation	no reflexes	no	Adrenaline, A	-
no	yes	intubation	no reflexes	no	Adrenaline	Adrenaline, A
no	yes	intubation	anaesthesia	no	Adrenaline, D	Adrenaline
no	yes	intubation	no reflexes	no	Adrenaline, Atropine	
no	no	intubation	anaesthesia	no	Ethomidate, I	-
yes	yes	intubation	no reflexes	no	Adrenaline	Adrenaline
no	no	no	-	no	-	NaCL
no	yes	intubation	no reflexes	no	Adrenaline, A	Catecholamir
no	yes	no	-	no	-	Adrenaline
no	no	oxygen face r	-	yes	NaCl, Ketami	Volume, FFP,
no	yes	intubation	no reflexes	no	Adrenaline, A	-
no	yes	intubation	no reflexes	no	Adrenaline, A	Vasopressin,
no	yes	intubation	no reflexes	no	Adrenaline, A	Adrenaline, T
no	yes	bag-valve-ma	-	no	-	Adrenaline, A
no	yes	intubation	no reflexes	no	Adrenaline, A	Adrenaline, A
yes	yes	intubation	no reflexes	no	Adrenaline, A	-
yes	yes	intubation	no reflexes	no	Adrenaline, A	Adrenaline
yes	yes	intubation	no reflexes	no	Adrenaline, A	Adrenaline
yes	yes	intubation	no reflexes	no	Adrenaline, A	Adrenaline
no	no	intubation	anaesthesia	no	Temesta	Dormicum, Fe
yes	yes	intubation	no reflexes	no	Adrenaline, A	Adrenaline
no	no	no	-	no	Voluven, NaC	-
no	yes	intubation	no reflexes	no	Adrenaline, A	Liquemin, Pla
yes	yes	no	no reflexes	no	Adrenaline, A	-
no	yes	intubation	no reflexes	no	Adrenaline, A	-
no	yes	intubation	no reflexes	no	Adrenaline, A	Catecholamir

no	no	no	-	no	-	Cefepime, Ko
yes	yes	intubation	no reflexes	no	Adrenaline, A	-
no	yes	intubation	anaesthesia	no	Adrenaline, A	Adrenaline
no	yes	intubation	no reflexes	no	Adrenaline	Adrenaline, A
no	yes	intubation	no reflexes	no	Adrenaline, A	-
yes	yes	intubation	no reflexes	no	Adrenaline, C	Adrenaline, A
no	yes	intubation	no reflexes	no	Dospir, Atrov	Adrenaline
yes	yes	intubation	no reflexes	no	Adrenaline, A	Adrenaline
no	no	no	-	no	-	Solumedrol, I
yes	yes	intubation	no reflexes	no	Adrenaline, A	Adrenaline
no	no	intubation	DM	yes	-	EK, FFP, Augn
no	yes	intubation	no reflexes	no	Ephedrin	-
yes	yes	intubation	no reflexes	no	Adrenaline, C	-
no	yes	intubation	no reflexes	no	Adrenaline, F	Atropine, Adr
no	yes	intubation	no reflexes	no	Glucose solut	Adrenaline, A
no	no	no	-	no	-	-
yes	yes	bag-valve-ma	-	no	-	-
no	no	no	-	no	-	Morphine
no	yes	intubation	no reflexes	no	Adrenaline, C	-
no	no	no	-	no	-	NaCL, Morph
no	yes	no	-	no	-	-
no	no	intubation	anaesthesia	no	-	-
no	no	no	-	no	-	Fentanyl, Pri
no	no	bag-valve-ma	-	no	-	-
yes	yes	intubation	no reflexes	no	Adrenaline, C	Adrenaline
no	yes	bag-valve-ma	-	no	-	Adrenaline
yes	yes	intubation	anaesthesia	no	Adrenaline, D	Adrenaline
no	no	no	-	no	-	-
yes	yes	intubation	no reflexes	no	Adrenaline	Adrenaline
no	yes	intubation	no reflexes	no	Adrenaline	-
yes	yes	intubation	no reflexes	no	Adrenaline	-
yes	yes	intubation	no reflexes	no	Adrenaline	-
yes	yes	intubation	no reflexes	no	Adrenaline, C	Adrenaline, A
no	yes	intubation	no reflexes	no	Adrenaline	Actilyse
yes	yes	intubation	no reflexes	no	Adrenaline	-
no	yes	intubation	anaesthesia	yes	Adrenaline, V	EK, FFP, TK, A
no	no	intubation	anaesthesia	no	Disoprivan, E	Adrenaline, N
no	yes	bag-valve-ma	-	no	-	Adrenaline
yes	yes	intubation	no reflexes	no	Adrenaline	-
no	yes	intubation	no reflexes	no	Adrenaline	-
no	yes	intubation	no reflexes	no	Adrenaline	FFP, EK, Cate
no	yes	intubation	no reflexes	no	Adrenaline	Naloxon, Flur
no	yes	intubation	no reflexes	no	Catecholamir	Adrenaline
yes	yes	intubation	no reflexes	no	Adrenaline	Adrenaline

yes	yes	intubation	no reflexes	no	Adrenaline	Adrenaline
yes	yes	DM	-	no	Adrenaline, A	Adrenaline
yes	yes	bag-valve-ma	-	no	Adrenaline, A	-
no	no	DM	-	no	ASS, Plavix	Liquemin, Pla
no	yes	intubation	no reflexes	no	-	Catecholamir
no	yes	intubation	no reflexes	no	Adrenaline, A	Adrenaline, A
no	yes	DM	-	no	Adrenaline	-
no	no	oxygen face r	-	no	-	Morphine
yes	yes	oxygen face r	-	no	Adrenaline	Adrenaline, A
yes	yes	intubation	no reflexes	no	Adrenaline, C	-
yes	yes	DM	-	no	Adreanlin	-
no	yes	intubation	no reflexes	no	Adrenaline, A	-
yes	yes	intubation	no reflexes	no	Adrenaline	-
no	yes	oxygen face r	-	no	-	Adrenaline
yes	yes	intubation	no reflexes	no	Adrenaline, C	-
no	yes	DM	-	no	unspecified C	-
yes	yes	LAMA	-	no	Adrenaline	-
yes	yes	intubation	no reflexes	no	Adrenaline	Adrenaline, C
no	yes	DM	-	no	Adrenaline	-
yes	yes	intubation	no reflexes	no	Adrenaline	-
no	yes	intubation	no reflexes	no	-	-
no	yes	intubation	no reflexes	yes	Adrenaline, N	Adrenaline
yes	yes	LAMA	-	no	Adrenaline, N	Adrenaline
yes	yes	DM	-	no	Adrenaline	Adrenaline, C
no	yes	intubation	no reflexes	yes	Adrenaline	-
yes	yes	intubation	no reflexes	no	Adrenaline	Adrenaline
no	no	no	-	no	-	-
no	no	intubation	anaesthesia	yes	-	FFP, EK
no	yes	intubation	no reflexes	no	Adrenaline	Adrenaline, V
no	yes	intubation	no reflexes	no	Adrenaline	-

ECG	ECG findings	Defibrillation	Imaging	Suspected cause of death
yes	PEA	no	no	cerebrovascular
yes	PEA	yes	DM	unclear cause of death
yes	ventricular fil	yes	no	pulmonary
yes	asystole	yes	no	suspected suicide
yes	tc sinus rhyth	no	X-ray thorax	cerebrovascular
yes	PEA, asystole	yes	no	suspected suicide
yes	ventricular fil	yes	no	cardiovascular
yes	tc sinus rhyth	no	X-ray thorax	neoplasia
yes	ventricular ta	no	no	cardiovascular
no	-	no	no	neoplasia
yes	ventricular fil	yes	no	cardiovascular
yes	PEA, ST segm	no	echocardiogr	cardiovascular
yes	PEA	yes	no	cardiovascular
yes	PEA	no	echocardiogr	unclear cause of death
yes	PEA, ROSC	yes	no	cardiovascular
yes	tc sinus rhyth	no	no	neoplasia
yes	asystole	no	no	suspected suicide
yes	ventricular fil	yes	no	cardiovascular
yes	PEA	yes	no	unclear cause of death
yes	ventricular fil	yes	no	cardiovascular
no	-	no	X-ray thorax,	cardiovascular
yes	PEA	no	FAST	accident
yes	ST segment d	no	sonography a	cardiovascular
no	-	no	no	neoplasia
yes	PEA	no	no	unclear cause of death
yes	PEA	no	no	cardiovascular
yes	PEA	no	no	accident
yes	ventricular fil	yes	no	accident
no	-	no	CT	neoplasia
yes	-	yes	FAST	cardiovascular
yes	asystole, ROS	no	echocardiogr	cardiovascular
no	-	no	sonography a	neoplasia
yes	ventricular fil	yes	no	cardiovascular
yes	ventricular fil	yes	no	cardiovascular
yes	ventricular fil	yes	no	cardiovascular
yes	PEA	no	no	cardiovascular
no	-	no	FAST, LODOX	suspected suicide
yes	nc sinus rhyth	no	FAST, X-ray th	cardiovascular
yes	PEA	no	FAST, CT	accident
yes	bc sinus rhyth	no	no	abdominal
yes	asystole, PEA	yes	sonography a	unclear cause of death
yes	PEA, tc sinus	no	CT	neoplasia
yes	Torsade de P	no	no	unclear cause of death

yes	PEA	no	FAST, X-ray th	abdominal
yes	-	no	CT	cerebrovascular
yes	asystole, vent	yes	no	unclear cause of death
yes	tc atrial fibrill	yes	no	cardiovascular
yes	ventricular fil	no	no	unclear cause of death
yes	asystole, ROS	no	no	unclear cause of death
yes	PEA	no	FAST	cardiovascular
yes	asystole	no	no	unclear cause of death
yes	PEA, ventricu	yes	echocardiogr	cardiovascular
yes	asystole	no	FAST	accident
yes	-	no	CT	cerebrovascular
yes	ventricular fil	yes	sonography a	cardiovascular
yes	PEA, ROSC, S	no	coronary ang	cardiovascular
yes	asystole	no	no	accident
yes	PEA, ventricu	yes	FAST, echoca	cardiovascular
yes	PEA	no	no	unclear cause of death
yes	ventricular fil	no	Lodox, FAST	suspected suicide
yes	ventricular fil	yes	no	cardiovascular
yes	asystole, vent	yes	no	cardiovascular
yes	ventricular fil	yes	no	unclear cause of death
yes	ventricular fil	yes	echocardiogr	cardiovascular
yes	PEA	no	no	unclear cause of death
yes	-	no	CT	cerebrovascular
yes	PEA, ventricu	yes	echocardiogr	unclear cause of death
no	-	no	CT	cerebrovascular
yes	AV Block 3, in	no	echocardiogr	cardiovascular
yes	nc sinus rhyth	yes	no	cardiovascular
no	-	no	Lodox, FAST,	suspected suicide
yes	ventricular fil	yes	echocardiogr	unclear cause of death
yes	ventricular fil	yes	echocardiogr	unclear cause of death
yes	PEA	no	echocardiogr	intoxication
no	-	no	no	cerebrovascular
yes	PEA	no	no	unclear cause of death
yes	asystole, PEA	no	no	cardiovascular
yes	ventricular fil	yes	no	cardiovascular
yes	asystole, PEA	no	echocardiogr	unclear cause of death
yes	PEA	no	no	cardiovascular
no	-	no	CT	cerebrovascular
yes	PEA, asystole	no	echocardiogr	unclear cause of death
yes	nc sinus rhyth	no	CT	cerebrovascular
yes	asystole, PEA	no	X-ray thorax,	cardiovascular
yes	ventricular fil	yes	no	cardiovascular
yes	asystole, ROS	no	no	cardiovascular
yes	ventricular fil	yes	echocardiogr	cardiovascular

no	-	no	X-ray thorax,	neoplasia
yes	PEA	no	echocardiogr	cardiovascular
yes	ventricular fil	yes	echocardiogr	unclear cause of death
yes	PEA	no	echocardiogr	cardiovascular
yes	ventricular fil	yes	no	unclear cause of death
yes	ventricular fil	yes	coronary ang	cardiovascular
yes	tc sinus rhyth	no	no	pulmonary
yes	asystole, PEA	yes	sonography a	cardiovascular
no	-	no	no	pulmonary
yes	ventricular fil	yes	no	cardiovascular
no	-	no	FAST, LODOX	suspected suicide
yes	PEA, atrial fib	no	no	cardiovascular
yes	ventricular fil	yes	no	unclear cause of death
yes	ventricular fil	yes	echocardiogr	unclear cause of death
yes	asystole	no	FAST	cardiovascular
no	-	no	no	neoplasia
yes	ventricular fil	yes	echocardiogr	unclear cause of death
yes	atrial fibrillati	no	no	cerebrovascular
yes	asystole, vent	yes	echocardiogr	unclear cause of death
yes	atrial fibrillati	no	X-ray thorax	cerebrovascular
yes	sinus rhythm,	no	CT	cardiovascular
yes	atrial fibrillati	no	CT, echocardi	cerebrovascular
yes	sinus rhythm	no	sonography a	cardiovascular
yes	tc sinus rhyth	no	no	cerebrovascular
yes	ventricular fil	yes	echocardiogr	unclear cause of death
yes	PEA, ventricu	yes	echocardiogr	cardiovascular
yes	PEA, ROSC	no	echocardiogr	cardiovascular
yes	tc sinus rhyth	no	CT	cardiovascular
yes	asystole, PEA	no	echocardiogr	unclear cause of death
yes	ventricular fil	yes	no	cardiovascular
yes	ventricular fil	yes	echocardiogr	unclear cause of death
yes	ventricular fil	yes	echocardiogr	unclear cause of death
yes	ventricular fil	yes	echocardiogr	cardiovascular
yes	PEA	no	echocardiogr	cardiovascular
yes	PEA, asystole	no	echocardiogr	unclear cause of death
yes	-	no	FAST, X-ray th	accident
yes	nc sinus rhyth	no	CT	cardiovascular
yes	PEA	no	echocardiogr	cardiovascular
yes	asystole	yes	echocardiogr	cardiovascular
yes	ventricular fil	yes	echocardiogr	unclear cause of death
yes	PEA, pacing r	no	sonography a	abdominal
yes	PEA, atrial fib	no	CT, sonograp	unclear cause of death
yes	asystole	no	no	unclear cause of death
yes	PEA, ROSC	no	echocardiogr	cardiovascular

yes	PEA, asystole	no	echocardiogr	unclear cause of death
yes	PEA	no	echocardiogr	cardiovascular
yes	ventricular fib	yes	echocardiogr	cardiovascular
yes	STEMI (inferio	no	echocardiogr	cardiovascular
yes	ST segment d	no	FAST, X-ray th	cardiovascular
yes	tc sinus rhyth	no	echocardiogr	cardiovascular
yes	asystole, vent	yes	no	unclear cause of death
yes	tc sinus rhyth	no	no	cardiovascular
yes	ventricular fib	yes	echocardiogr	cardiovascular
yes	ventricular fib	yes	no	unclear cause of death
yes	PEA, asystole	no	no	cerebrovascular
yes	PEA, asystole	no	echocardiogr	unclear cause of death
yes	ventricular fib	yes	no	cardiovascular
yes	sinus rhythm,	no	echocardiogr	cardiovascular
yes	ventricular fib	yes	echocardiogr	cardiovascular
yes	ventricular fib	yes	echocardiogr	cardiovascular
yes	ventricular fib	yes	no	unclear cause of death
yes	ventricular fib	yes	echocardiogr	cardiovascular
yes	asystole, ROS	yes	no	unclear cause of death
yes	asystole	yes	echocardiogr	unclear cause of death
yes	PEA	no	no	neoplasia
yes	PEA, asystole	no	FAST	unclear cause of death
yes	asystole	no	no	unclear cause of death
yes	asystole	yes	no	unclear cause of death
yes	asystole, PEA	no	FAST, echoca	accident
yes	asystole	no	echocardiogr	cardiovascular
no	-	no	no	cardiovascular
no	-	no	FAST, CT	accident
yes	PEA, ventricu	yes	FAST, echoca	cardiovascular
yes	asystole, vent	yes	FAST, echoca	unclear cause of death

**Use of Lucas™"**
no
no
no
no
no
no
no
no
no
no
no
no
no
no
no
no
no
no
no
no
no
no
no
no
no
no
no
no
no
no
no
no
no
no
no
no
no
no
no
no
no
no
yes

no
no
no
no
no
no
no
no
no
no
no
no
yes
no
no
no
no
no
no
no
no
no
no
no
no
no
no
no
no
no
no
no
no
yes
no
yes
yes
no
no
no
yes
yes
yes
no

no
yes
no
no
no
yes
no
yes
no
no
no
no
yes
yes
no
no
yes
no
no
no
no
no
no
no
yes
no
yes
no
yes
no
yes
yes
yes
yes
yes
no
no
yes
no
yes
no
yes
yes
yes

yes
yes
yes
no
no
yes
no
no
yes
no
no
yes
yes
no
yes
no
yes
no
yes
yes
no
yes
yes
yes
yes
yes
no
no
yes
yes
