# Supplementary material for: The dead and the dying - a difficult part of EMS transport: A Swiss cross-sectional study
Source: PLoS One. 2018 Feb 27;13(2):e0191879. doi: 10.1371/journal.pone.0191879 (PMC5828436; doi:10.1371/journal.pone.0191879)
Supplement: S2 File — (PDF) [file pone.0191879.s002.pdf]

Legend Minimal Data Set

- ED = emergency department
- SBP = systolic blood pressure
- DBP = diastolic blood pressure
- SpO2 = peripheral oxygen saturation
- GCS = Glasgow Coma Scale
- NACA-Score = National Advisory Committee for Aeronautics score
- CPR = cardiopulmonary resuscitation
- ECG = electrocardiogram
- Spine Management = including spine board or stiff neck protector
- Lucas™2 = mechanical chest compression device
- 9999 = no detectable parameter
- DM = data missing
- PEA = pulseless electrical activity
- ROSC = return of spontaneous circulation
- STEMI = ST-segment elevation myocardial infarction
- tc = tachycardiac
